# Physical Activity Effects on Muscle Fatigue in Sport in Active Adults with Long COVID-19: An Observational Study

**DOI:** 10.3390/diagnostics13071336

**Published:** 2023-04-03

**Authors:** Francesco Coscia, Rosa Mancinelli, Paola Virginia Gigliotti, Franco Checcaglini, Giorgio Fanò-Illic

**Affiliations:** 1 Sports Medicine Service of the San Candido, Innichen and Brunico-Bruneck Hospitals, Bolzano-Bozen, 39038 San Candido, Italy; francesco.coscia1@gmail.com; 2 Department of Neuroscience Imaging and Clinical Sciences, University “G. d’Annunzio” of Chieti-Pescara, 66100 Chieti, Italy; r.mancinelli@unich.it; 3 IIM-Interuniversity Institute of Myology, University “G. d’Annunzio” of Chieti-Pescara, 66100 Chieti, Italy; 4 Laboratory of Sport Physiology San Candido-Innichen, University of Perugia, 39038 San Candido, Italy; pao.gigliotti@gmail.com; 5 Campus of Free University of Alcatraz, Free University of Alcatraz, Santa Cristina di Gubbio, 06024 Gubbio, Italy; franco.checcaglini@libero.it; 6 A&C M-C Foundation for Translational Myology, 35100 Padova, Italy

**Keywords:** long COVID-19 syndrome, myalgic encephalomyelitis/chronic fatigue syndrome, (EM/CFS), physical exercise, muscle fatigue, mild cognitive impairment, sleep quality

## Abstract

Long COVID-19-related changes in physiology includes alterations in performing muscle work as fatigue. Data available do not allow us to define the usefulness of physical activity to attenuate long COVID-19 functional modifications. The present observational study investigates the effects of physical activity on the perception of fatigue, maximum power output, sleep, and cognitive modifications in subjects affected by long COVID-19, distinguishing between active and sedentary subjects. The data demonstrated the following: the perception of fatigue 1 year after the end of virus positivity was significantly reduced with respect to that observed after 6 months by more than 50% more in active subjects compared to sedentary ones; 6 months after the end of virus positivity, the force developed by active subjects was reduced (RM factor: *p* < 0.001, η2p = 0.527, post hoc: *p* < 0.001), but the reduction was more pronounced in sedentary ones (mean difference = 38.499 W); poor sleep quality and mild cognitive impairment were assessed in both active and sedentary subjects. In conclusion, the study suggests that the long COVID-19 fatigue was lower in active subjects respect to sedentary ones. A comparative analysis performed due to the overlap of functional alterations between long COVID-19 and ME/CFS showed that in a small percentage of the enrolled subjects (8%), the symptomatology reflected that of ME/CFS and was independent of the individual physical capacities.

## 1. Introduction

The clinical definition of long COVID-19 is used to characterize the modification of some functional aspects of individuals who have passed the acute phase of infection and have returned a negative result on molecular control tests. The status of long COVID-19 refers to situations wherein patients report persistent symptoms, i.e., those that persist for at least 60 days after diagnosis or for at least 30 days after recovery from acute illness or discharge from hospital [1]. In general, and in the absence of an international standard defining them, the long-lasting effects of infection occur after patients have presented with the usual clinical pattern of the disease for much longer than expected [2]. Aspects of long COVID-19 result in two different symptomatic and prognostic types: (a) a serious form, due to the occurrence of thromboembolic complications, and (b) a milder form, characterized mainly by muscle fatigue and dyspnea [3].

Initial studies of some significance provided some thoughts for the identification of the risk-to-patient ratio for the onset of long COVID-19 and for the identification of persons at high risk of post-acute COVID-19 [4]. The first finding concerned the severity of the disease during the acute phase [5]. In these patients, the possibility of onset of long COVID-19 syndrome was characterized by the presence of symptoms such as dyspnea and muscle fatigue/weakness [6]. Other comorbidities, such as diabetes, obesity, cardiovascular disease, or cancer, which are determinants of increased severity and mortality related to the acute phase of COVID-19, did not appear to be associated with the onset of long COVID-19 [7]. A large meta-analysis conducted in the U.K. included risk factors associated with the duration and severity of symptoms present during the long COVID-19 phase, with a focus on age, female gender, and the presence of respiratory and metabolic pathologies. Regarding ethnic group membership, Whites were associated with a higher risk of long COVID-19 than Asians and Blacks [8].

A recent study conducted in Italy reported the persistence of symptoms in 87.4% of patients discharged from hospital after acute COVID-19; fatigue (53.1%), dyspnea (43.4%), joint pain (27.3%), and chest pain (21.7%) were the most commonly reported symptoms [9]. Even in a broader meta-analysis, applied to a cohort of approximately 250,000 individuals, it was highlighted that the most common and protracted symptoms (lasting up to or more than 12 months past the end of the acute phase) showed a prevalence toward fatigue, dyspnea, sleep disturbance, and myalgia (41%, 31%, 30%, and 22%, respectively) [10].

Based on the synthesis of studies reviewed so far, it seemed clear that fatigue was the most frequently reported symptom in long COVID-19 patients. In this scenario, the need for monitoring and treatment for post-COVID-19 patients is evident. Advancements and standardization of long COVID-19 research methodologies would improve the quality of future research [11]. Recently, a virtual meeting of the Physiological Society (U.K.), held in February 2022 and published in January 2023, brought together clinicians and researchers to take stock of risk factors, therapies, and recovery deadlines for long COVID-19 sufferers, highlighting some important aspects of this pathology. A first consideration that can be made is that long COVID-19 research has been most effective in those populations that routinely monitor their physical performance, namely, military and elite athletes [12]. The results obtained on these subjects significantly highlight the success of the anti-long COVID-19 strategies implemented. This, of course, also has a positive spin-off for treatments to be applied to the general population, highlighting how the high degree of success of diagnosis, intervention, and monitoring in these active populations may suggest strategies to be applied to others.

Myalgic encephalomyelitis, or chronic fatigue syndrome (ME/CFS), is a complex, multisystem disorder that involves debilitating symptoms, including profound fatigue, nonrestorative sleep, cognitive deficits, and/or orthostatic intolerance [13]. The onset of the typical symptomatology of ME/CFS [14] led to the clinical diagnosis of the disease, as established by the Committee on the Diagnostic Criteria for post-infectious fatigue syndromes [15]. Several stressors can worsen all disease symptoms, a condition called post-stress malaise (PEM) [16]. Since the most prominent symptom of ME/CFS is significant difficulty in performing even very mildly loaded exercise, the use of exercise therapy has been attempted in recent decades to try to alleviate this problem [17]. The only effective strategy related to physical activity in ME/CFS patients is so-called pacing, a limitation of both physical and metabolic activities [12,13]. At some extent, long COVID-19 and ME/CFS show an overlap of symptoms [18,19]. In a recent paper, Wright et al. tried to measure the effects of exercise on long COVID-19 sufferers but did not reach an uncontroversial consideration. Since nearly 80% of the study participants reported experiencing a worsening effect, while only less than 1% reported improvement, the conclusion was that, like ME/CFS syndrome, physical activity programs in long COVID-19 patients worsened the patient’s clinical situation [20].

In the present study, young active adults (competitive, amateur, and professional athletes) were enrolled and observed up to one year after the end of COVID-19 positivity and compared with sedentary people with similar anthropometric characteristics. In particular, some functional capacities such as muscle strength and fatigue, sleep characteristics, and cognitive modifications were assessed in order to determine the capacity of individuals to recover pre-COVID-19 fitness status.

In addition, a comparative analysis performed due to the overlap of functional alterations between long COVID-19 and ME/CFS was performed to clarify the efficacy of physical activity programs on the duration and intensity of long COVID-19 muscle symptoms without leading to the detrimental effects highlighted in patients with ME/CFS.

## 2. Materials and Methods

Recruitment (*n* = 506, of which 138 were females) was carried out on a population of approximately 6000 subjects who had come for observation at the Sports Medicine Service of the Hospitals of San Candido-Inniken and Brunico-Brunek in the Autonomous Province of Bolzano-Bozen (Italy). Due to the proximity to the Dolomites mountain range, residents were primarily skiers. The population enrolled consisted of active subjects, even with some differences in terms of prevalence of aerobic or anaerobic activity, who had performed an incremental stress test in the year prior to infection. Indeed, based on the type of physical activity and/or sport practiced, the enrolled subjects were divided into the following four categories:

1. Competitive cross-country ski athletes (AA),
2. Mountain amateurs (MA),
3. Ski instructors (SI),
4. Sedentary people (SP).

The last category represents the control group.

All enrolled subjects presented, six months after the end of the COVID-19 positive period, marked signs of muscle fatigue.

The enrolled subjects had been affected by COVID-19 previously (June 2020–October 2021) and had been negative in the molecular test for at least 6 months. None of them had required hospital treatment during the period of illness. Before the formation of the groups, participants were informed of all procedures related to the observational study, given full instructions, and asked to provide written informed consent. The study was subject to institutional ethical approval and carried out in accordance with the Declaration of Helsinki. The anamnesis of each subject was documented, and general health status was assessed. Body height and weight were measured manually (SEKA mod 701, Japan). In addition, a 12-lead electrocardiogram (ECG) was recorded for each subject (Stress ECG COSMED, Italy). Blood pressure was also measured manually at this stage.

**Figure 1:**
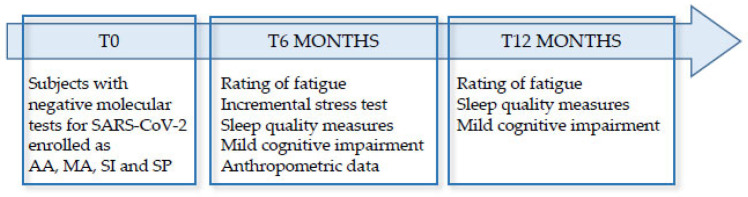
Schematic illustration of the study design. Subjects enrolled were tested as described in the boxes at 6 and 12 months after the end of COVID-19 acute infection.

Eligibility criteria included male or female individuals aged ≥20; ≤40 years with a body mass index (BMI) of 18.0–29.9 kg/m^2^. Participants had to be metabolically healthy and normoglycemic after an overnight fast. Systolic and diastolic blood pressure had to be in the range of 95–150 and 60–85 mmHg, respectively. In addition, taking any medication with a potential impact on the assessed parameters (blood pressure lowering therapy, anti-arrhythmic drugs, antidepressants, etc.) was classified as an exclusion criterion. All enrolled subjects, six months after the end of the COVID-19 positive period, were tested for: rating of fatigue, incremental stress test, sleep quality measures, mild cognitive impairment, and anthropometric data; the rating of fatigue was measured again 12 months after the end of the COVID-19 positive period in all subjects. The study design is shown in Figure 1.

### 2.1. Rating of Fatigue (ROF)

The quantitative perception of fatigue was measured 6 and 12 months after the end of the COVID-19 positive period according to the ROF scale, with values between 1 and 10. This method has demonstrated good validity for incremental tests, as well as for recovery at rest and activities of daily living. The intensity-based approach to measuring perceived fatigue adopted with the ROF scale appeared to support theoretical notions that perceived fatigue should be considered a global perceptual phenomenon [21].

### 2.2. Incremental Stress Test

Incremental stress test (modified Bruce cycle ergometer test) was performed on a variable-resistance cycle ergometer (COSMED Srl, Rome, Italy), with the subject connected for continuous ECG recording. After a no-load warm-up phase of 1 min, the test began with a load of 25 watts, again for 1 min, which was followed by the incremental resistance phase (50 watts every 2 min). A physician constantly monitored each patient’s cardiac activity, AP, and clinical sporting status, according to the progressive load automatically delivered by the program. After 10 min, the cooldown phase began (1 min at 50 watts, 2 min at 25 watts, and again, 2 min without load) until the initial physiological characteristics were restored [22].

### 2.3. Sleep Quality Measures

The evaluation of sleep disturbances—assessed exclusively as the presence of poor sleep quality—was derived from the administration of the PSQI (Pittsburgh Sleep Quality Index) to participants. The PSQI questionnaire classically consists of 9 items that form the basis of the score plus 5 items that concern the sleeping partner. The self-assessment test we delivered included the reply to the 9 items but not the subgroup analysis that many items predict by eliminating the component concerning the partner given the age of the sample. The PSQI is a rating scale capable of providing a reliable, valid, and standardized measure of sleep quality. Poor sleep quality was defined as a global PSQI score > 5 [23].

### 2.4. Mild Cognitive Impairment

The presence of problems related to memorization processes and difficulties in concentrating can be assessed through internationally validated tests. The Mini–Mental State Examination (MMSE), or Folstein test [24], is a gold standard that is used extensively in clinical and research settings to measure cognitive impairment. The test battery is composed of thirty items, referring to seven different cognitive areas, but we used a short (nonstandard) version of it (prepared by us). Only five areas (time perception, spatial orientation, word registration, attention and computation, and language) were used in the analysis. Subjects who did not reach at least 75 percent of the maximum score were considered deficient in order to highlight the presence of mild cognitive alterations.

### 2.5. Statistical Analysis

GraphPad Prism 5.0. software (GraphPad Software San Diego, CA, USA) was used to plot graphs. Statistical analyses were carried out on Jamovi software (version 2.3.18.0). Before proceeding with the comparisons, the Shapiro–Wilk test for normality of the distributions was checked and, along with comparisons, the Levene test for homogeneity of variances and Q-Q plots were evaluated. Comparisons were conducted with a repeated-measures ANOVA, setting pre vs. post as the within factor, and group and sex as between factors. The Tukey correction for post hoc comparisons was applied. Partial eta squared (η2p) was computed as effect size.

## 3. Results

Based on the type of physical activity and/or sport practiced, the enrolled subjects were divided in the following four categories:

1. Competitive cross-country ski athletes (AA),
2. Mountain amateurs (MA),
3. Ski instructors (SI),
4. Sedentary people (SP).

The selected statistical sample (about 8% of the whole population tested) consisted of young adults with an average age of approximately 33 years who were divided into four categories. The first category consisted of competitive cross-country ski athletes (AA). Due to the type of sport they practiced (cross-country skiing), the physical training of the AA group could be defined as predominantly aerobic. The second group, those defined as mountain amateurs (MA), consisted of a heterogeneous group of participants devoted to various types of mountain sports; however, these were practiced discontinuously and never competitively. The third group, with relatively high physical capacity, was ski instructors (SI); in these subjects, the training for their activity was mixed with anaerobic predominance. The last category, composed of individuals who did not practice continuously any specific motor activity, was defined as sedentary people (SP).

Anthropometric data are shown in Table 1 and revealed the highly homogeneous nature of the total sample, which varied only in numerosity.

The average age of the four categories meant that all the subjects analyzed fell into the young adult group. BMI data showed that all subjects analyzed were also in the normal range at the time the experimental study started.

In order to highlight both the presence of sleep disturbances and mild cognitive impairments, PSQI and MMSE modified questionnaires were submitted to subjects recruited.

The results are shown in Table 2 as the percentage of the presence of poor sleep quality and/or mild cognitive impairments in the four categories.

**Figure 2:**
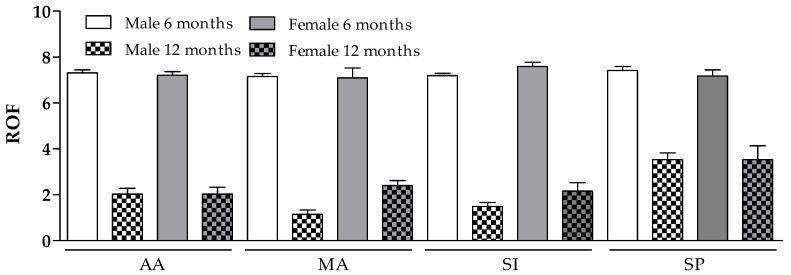
Rating of Fatigue (ROF) analysis. In almost all pairwise comparisons, the values at 12 months are markedly lower than those at 6 months. Considering that assumption checks were violated, RM-ANOVA is not allowed. The descriptions show that the reductions at 12 months are less marked in the SP group (mean difference of 3.6 for females and 3.9 for males) than in the AA (mean difference of 5.2 for females and 5.3 for males), MA (mean difference of 4.7 for females and 6 for males), and SI groups (mean difference of 5.4 for females and 5.7 for males).

Figure 2 shows the Rating of Fatigue (ROF) analyzed 6 and 12 months after the end of the anti-COVID-19 molecular buffer positivity period. In general, data showed that, in almost all pairwise comparisons, the values at 12 months were markedly lower than those at 6 months. Considering that the assumption checks were violated, RM-ANOVA was not allowed. At the end of the first six-month period, all enrolled subjects, whatever category they belonged to, revealed a fatigue rating, as measured by the ROF scale, greater than seven, with no significant differences between groups. However, when the same type of analysis was performed at the end of the subsequent six months, the results changed. While the perception of fatigue was reduced by about 50% in the sedentary people group (with no gender difference), in the other three categories (representing subjects practicing constant physical activity, albeit at different levels of intensity), the reduction was significantly higher. Indeed, the descriptions showed that the reductions at 12 months were less marked in the SP group (mean difference of 3.6 for females and 3.9 for males) than in the AA (mean difference of 5.2 for females and 5.3 for males), MA (mean difference of 4.7 for females and 6 for males), and SI groups (mean difference of 5.4 for females and 5.7 for males).

Interestingly, less than 10% of the statistical sample, with no variation between genders and categories, had persistent muscle fatigue symptoms greater than or equal to 5 as ROF, one year after the end of the acute disease. Mild impairment of cognitive processes and lack of restorative sleep was also present in this subpopulation (Table 3).

In summary, in only 36 subjects out of the 506 covered by this observational study, the typical prerequisites of myalgic encephalitis/CFS were present at the same time (Table 3).

**Figure 3:**
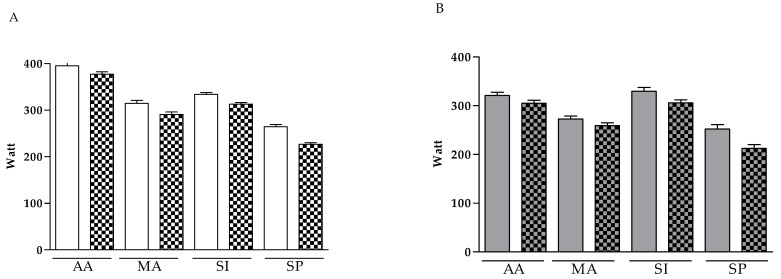
Incremental test. Statistical analyses were carried out on Jamovi software (version 2.3.18.0). Panel (**A**) refers to the data obtained in males; panel (**B**), in females. Empty bars refer to pre-COVID-19 while dotted bars refer to post-COVID-19. Before proceeding with the comparisons, the Shapiro–Wilk test for normality of distributions is performed. Along with comparisons, the Levene test for homogeneity of variances and Q-Q plots are evaluated. Comparisons are conducted with a repeated ANOVA measure, setting Pre vs. Post as the within factor, and group and sex as between factors. The Tukey correction for post hoc comparisons was applied. Partial eta squared (η_2_p) was computed as effect size. *p*< 0.001 in all comparison.

The data shown in Figure 3 (panels A and B) refer to the power (watts) recorded during 10 min incremental stress testing on the subjects enrolled in the experimental study. Figure 3 panel A refers to data obtained for males, while panel B reports the measurements obtained in females.

The data refer to the test carried out six months after the end of the disease in the presence of an average ROF score higher than 7. The results of the post-COVID-19 test were compared with those calculated directly by the software and defined as maximal based on the characteristics of the subject examined. As can be seen from the graph, the values recorded in males were always higher than those recorded in females, as expected.

Statistical analysis of the data showed that watts at post were reduced, with respect to pre, in all groups (RM factor: *p* < 0.001, η2p = 0.527, post hoc: *p* < 0.001); additionally, there was a difference between the groups in the trend from pre to post (RM × group: *p* < 0.001, η2p = 0.121). In particular, in the group of Sedentary People, despite a lower starting level than the others, the reduction was more pronounced (mean difference = 38.499 W) in comparison with all other groups (AA = 17.238 W, MA = 23.078 W, SI = 21.655 W). Among the three groups, the AA group seemed to be the most protected, by the marked reduction of watts at post. This result was not influenced by gender (RM × group × gender: *p* = 0.120, η2p = 0.012).

## 4. Discussion

The first objective of the observational study described in this paper was to define—in a population consisting of young adult subjects, homogeneous in lifestyle with an above-average degree of physical activity—the presence of long COVID-19 symptomatology as that described the general population [25]. It is important to note that this status has been described, not only in adults but also in pediatric and adolescent patients [26]. Furthermore, it is worth noting that, at least with regard to some biomarkers related to training load, such as the presence of TnC in the blood, there seems to be a manifest change in adolescent sports subjects as a consequence of the physical exercise (soccer) undertaken [27]. Vice versa, the presence of previous SARS-CoV-2 infection does not significantly change this pattern in elite soccer athletes [28]. In addition, it has occurred among patients with severe symptoms, but has also been reported regardless of the severity of the acute phase, hospitalization, and so on [29].

The starting point for achieving this was the formation of a statistically congruous sample, according to the internationally known parameters for the definition of long COVID-19 syndrome, i.e., the presence of a persistent state of muscle fatigue, sometimes associated with episodes of dyspnea, due to the presence of unresolved states of stress, mild cognitive impairment (memory and attention), and/or sleep disturbance [7,30].

As shown in Figure 2, it was possible to show, in all enrolled subjects, six months after the end of the acute phase of COVID-19, the presence, on the ROF scale, of a very high mean value of the perception of fatigue (almost 8, on a scale ranging from 1 to 10) in all tested categories, without gender differences. A recent paper [31] tested the existence of a long COVID-19 phase in patients discharged from Wuhan Hospital but who had not had major lung impairment. At 6 months after the end of the acute infection, the subjects primarily exhibited problems with fatigue or muscle weakness, sleep management difficulties, and anxiety or depression. Although the selected population differed in age, lifestyle and likely disease severity, the data from our study were somewhat comparable with the cohort of Wuhan patients. This situation changed significantly when the same observation was made one year after the end of the acute phase of the disease. Under these conditions, while the three categories with higher motor efficiency (AA, SI, and MA) revealed an ROF score close to 2, the sedentary people group had a value about twice as high. If one wanted to draw a general conclusion from these first data, one would have to assume that physical capacity and a more active lifestyle were able to significantly reduce the major and most debilitating symptom of long COVID-19 syndrome.

On the other hand, the positive value of physical activity in debilitating situations for the body, senescence, or other syndromes, even of viral origin, has been widely demonstrated [32,33,34].

One of the main objectives of this observational research project was to see if there were any similarities between the symptomatology present in long COVID-19 syndrome and that in patients suffering from a disease such as ME/CFS, in which the predominant symptom is the presence of an intense perception of fatigue that cannot be resolved by rest [9]. Attempts made, even in recent years, to define the impact of physical activity as a therapy, at least adjuvant, in patients suffering from MC/CFS, have highlighted that contradictory results worsen patients’ capacity to adequately perform daily activities. In fact, as indicated by the Cochrane Database, the use of an established degree of activity modulated over time increased the patients’ capacity to have an almost normal life [35]. On the other hand, the use of other strategies, such as pacing, which is a strategy to manage the availability of energy to complete activities of daily living, can improve the clinical situation of patients. According to these authors, in most patients, therapy based on continuous and graded exercise led to worsening of symptoms. Meanwhile, cognitive-behavioral therapy led to no change in symptoms, and pacing led to improvement [36,37].

The effectiveness of exercise therapy seemed greater than that of pacing; however, like cognitive treatment, it was a supportive therapy. Randomized trials with a low risk of bias are needed to investigate the type, duration, and intensity of the most beneficial exercise intervention [38].

Paraphrasing what was written by Simon Dècary et al., “The history of ME/CFS with exercise is a story of false hope. More than 3 decades of exercise attempts in this population can be summed up in one sentence: exercise can be harmful, sometimes life-threatening, and should be avoided” because it causes the onset of a post-exertional malaise called PEM. PEM is an abnormal physiological response to physical or cognitive stress that causes a severe combination of parainfluenza and neurological symptoms and, most importantly, overwhelming fatigue [39].

From the data we were able to observe, the selected subjects displayed, after a period of six months from the end of the acute phase of COVID-19, a very high rate of fatigue associated with other symptoms such as memory and/or attention disorders. Most importantly, in a large percentage, the presence of non-restorative sleep was observed. All of this was noted without any differences in either gender or undertaken physical activity. For this reason, a superficial analysis of the data seemed to indicate a close similarity between long COVID-19 status and ME/CFS syndrome, as indicated by many authors [40].

However, continuing the prospective observation up to one year after the end of the acute phase of the disease, the situation seemed radically changed. The perception of fatigue was drastically reduced in all groups, but with a significant difference in the more historically active groups (AA, SI, and MA) compared with sedentary people. During this phase only, a slight gender difference was observed in the motor-active groups. Nevertheless, only a longer period of observation will allow us to determine whether the phenomenon of fatigue may recur and in what manner. This last observation does highlight a key difference in the situation between long COVID-19 and ME/CFS. In the tested sample, consisting of mature young people with a high degree of physical efficiency, the symptomatology typical of ME/CFS could be considered transient.

However, it should be kept in mind that, one year after the end of the acute phase of COVID-19, in a small fraction of the tested population (36 out of 516 individuals), the perception of a state of fatigue that could not be quickly resolved with appropriate rest after even non-exhaustive motor activity was still present. Members of this subpopulation also manifested mild cognitive impairment and stated significant sleep disturbance [41].

**Figure 4:**
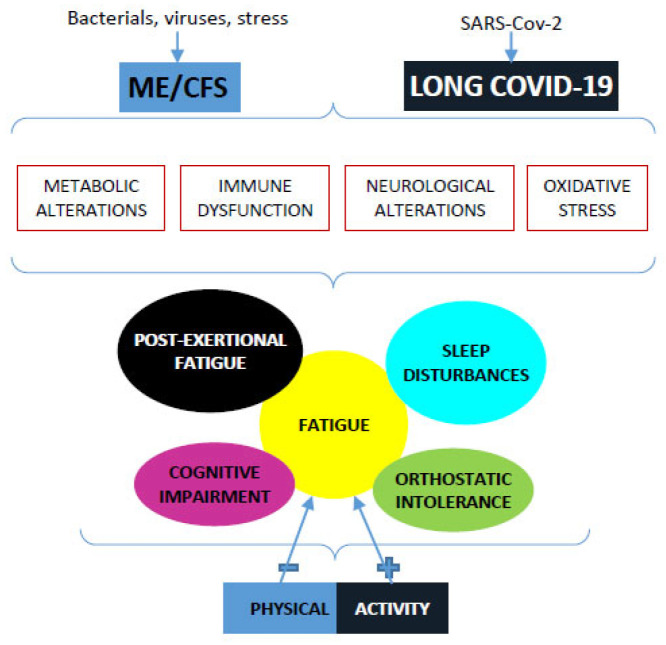
Similarities between ME/CFS and long COVID-19. Model depicting potential mechanisms involved in ME/CFS and long COVID-19. The combined effect of bacterial/virus insults and stress can trigger neurological, immune and metabolic dysfunction, and oxidative stress, which together could contribute to the symptomology observed in ME/CFS and long COVID-19 patients. Physical activity may be beneficial in the management of long COVID-19 but with more attention in ME/CFS patients.

In this population, which we followed in a prospective observational study, the similarities between ME/CFS (but not only) and long COVID-19 seemed to be present, although the small sample size did not allow us to reach conclusive confirmation [42] (Figure 4). For those manifesting this condition, the proposal made by Simon Decary et al. in the above-cited editorial, STOP-REST-PACE, could find the right application.

## 5. Conclusions

In conclusion, our observational study was carried out in young adults undertaking constant motor activity in a competitive and non-programmed manner. In this population, the duration and intensity of fatigue sensation related to the presence of long COVID-19 were less than those recorded in sedentary people. For this reason, we came to the belief that physical activity, or rather familiarity with physical activity, could be a favorable prognostic factor for the alleviation of long COVID-19-related symptoms. A prospective study is underway at the time of writing to highlight any statistically significant correlations between physical activity levels and the duration and intensity of long COVID-19-related symptoms.

Finally, it seemed likely to us, at least in the sample we tested, that the similarities between long COVID-19 and ME/CFS were apparent and, above all, transient. The duration of the perception of functional alterations appeared to be present one year after the end of infection only in a small percentage of the population examined (about 8%). However, this subpopulation is currently being observed; no update can be reported before the end of 2023.

## Figures and Tables

**Table 1:** Anthropometric characteristics of the enrolled subjects.

	AA-m	AA-f	MA-m	MA-f	SI-m	SI-f	SP-m	SP-f
Numerosity	115	66	133	45	75	10	45	17
Age (Years)	28 ± 5.4	32.9 ± 5.2	31.8 ± 4.7	32.6 ± 6	33.2 ± 4.6	32.9 ± 6.3	35 ± 3.8	33.6 ± 4.9
Height (cm)	178.2 ± 0.8	161.8 ± 3	175.8 ± 5.6	172.2 ± 9.7	172.8 ± 9.4	172.2 ± 10.6	170.8 ± 7	167.4 ± 10.8
BMI	22.8 ± 2	21.9 ± 0.8	24.1 ± 2.6	21.4 ± 2.6	22.4 ± 3.8	21.4 ± 4.1	20 ± 2.4	20.9 ± 2.7

Anthropometric data are presented as mean ± standard deviation. m—male gender; f—female gender.

**Table 2:** Percentage of the presence of poor sleep quality and mild cognitive impairment in enrolled subjects.

	AA-m	AA-f	MA-m	MA-f	SI-m	SI-f	SP-m	SP-f
Poor sleep quality	43.8	56.3	65.3	50	60.1	60.0	48.8	58.8
Mild cognitive impairment	31.3	46.1	51.3	50	38.6	50.4	36.4	37.5

Data are presented as percentage. m—male gender; f—female gender.

**Table 3:** Percentage, by category, of the simultaneous presence of mild cognitive impairment, poor sleep quality, and persistent muscle fatigue one year from the end of COVID-19 presence.

	AA-m	AA-f	MA-m	MA-f	SI-m	SI-f	SP-m	SP-f
Number	11	7	2	1	8	2	2	1
Percentage	9.3	10.7	4.5	10	6	6.8	4.5	6.25
ROF 6 months	9.6	9	9.5	9	8.5	7	8.7	9
ROF 12 months	7	6.2	7	5	6.2	5.5	6.7	7

Number and percentage of compresence of mild cognitive impairment and poor sleep quality.

## Data Availability

The data presented in this study are available on request from the corresponding author. The data are not publicly available due to policy to defend the privacy of sensitive data of the participating hospitals.
